# Angiogenic regulation of dental pulp stem cells

**DOI:** 10.3389/fdmed.2025.1717150

**Published:** 2025-12-11

**Authors:** Waruna Lakmal Dissanayaka, Nunthawan Nowwarote, Tanida Srisuwan, Sirawut Hiran-Us, Chatvadee Kornsuthisopon, Xiaofei Zhu, Thanaphum Osathanon

**Affiliations:** 1 Applied Oral Sciences & Community Dental Care, Faculty of Dentistry, The University of Hong Kong, Hong Kong, Hong Kong SAR, China; 2 Université Paris Cité, INSERM UMR1163, Imagine Institute, Paris, France; 3 Department of Oral Biology, Faculty of Dentistry, Université Paris Cité, Paris, France; 4 Department of Restorative Dentistry and Periodontology, Faculty of Dentistry, Chiang Mai University, Chiang Mai, Thailand; 5 Department of Operative Dentistry, Faculty of Dentistry, Chulalongkorn University, Bangkok, Thailand; 6 Centre of Excellence for Dental Stem Cell Biology and Department of Anatomy, Faculty of Dentistry, Chulalongkorn University, Bangkok, Thailand; 7 Department of Endodontics, Henry M. Goldman School of Dental Medicine, Boston University, Boston, MA, United States

**Keywords:** angiogenesis, dental pulp, hypoxia, growth factors, VEGF

## Abstract

Tissue regeneration relies on the ingrowth of blood vessels from the host for the survival and functionalization of regenerated tissues. Any holdup in this process can threaten the viability of the transplanted progenitor cells, which in turn can hinder effective tissue regeneration. Dental pulp stem cells (DPSCs) are a promising candidate cell source for dental pulp regeneration due to their potential for odontogenic and endothelial differentiation, as well as angiogenic properties. This narrative review examines the mechanisms by which DPSCs regulate angiogenesis. DPSCs modulate angiogenesis through multiple mechanisms: direct differentiation into endothelial cells, paracrine secretion of angiogenic growth factors, and functioning as mural cells to stabilise the nascent vasculature formed. Furthermore, the physical and biological interaction between DPSCs and extracellular matrices modulate the process of angiogenesis. The primary focus is on the intricate, multifaceted aspects of dental pulp regeneration; however, broader aspects of general tissue regeneration were also highlighted. The angiogenic modulation by DPSCs holds significant potential for the formulation of strategies that integrate pro-angiogenic scaffolds and signalling molecules to address the challenges associated with dental pulp tissue regeneration.

## Introduction

Angiogenesis and vasculogenesis play essential roles in the survival and development of transplanted cells during tissue regeneration (1). This aspect is particularly significant in dentin-pulp regeneration due to the limited potential for receiving a host-derived blood supply to the entire root canal system, constrained by the unique anatomy of the tooth, which features a small opening at the apical foramen (less than 0.3–1 mm), permitting limited microvasculature.

Dental pulp stem cells (DPSCs) are a promising candidate cell source for dental pulp regeneration for their odontogenic and endothelial differentiation potential as well as angiogenic properties. Specific perivascular cells have been found to positively stain for markers like STRO-1 (mesenchymal cell marker), CD146 (endothelial cell marker), and α-smooth muscle actin (marker for pericytes), which DPSCs are also positive for, suggesting phenotypic similarities (2, 3). Accordingly, DPSCs play a pivotal role in maintaining the perivascular niche and exhibit angiogenic properties.

Being located in a perivascular niche (2, 4), the functional crosstalk between DPSCs and endothelial cells (ECs) is well-documented; both cell types can induce pro-angiogenic effects on one another. EC function extends beyond merely being part of the vascular supply that facilitates oxygen and nutrient influx. They also contribute to preserving populations of stem cells throughout the lifespan of the dental pulp through paracrine pathways (4). ECs release IL-6, bind to IL-6R on DPSCs, activating STAT3 signalling and promoting B-cell-specific Moloney murine leukemia virus Integration site 1 (Bmi-1) expression, essential for maintaining perivascular niches within the dental pulp (4). Thus, DPSCs can create perivascular niches through asymmetric division. This evidence further strengthens the pro-angiogenic nature of DPSCs, while many subsequent studies demonstrated the paracrine signalling that mediates DPSC-mediated angiogenesis.

**Figure 1:**
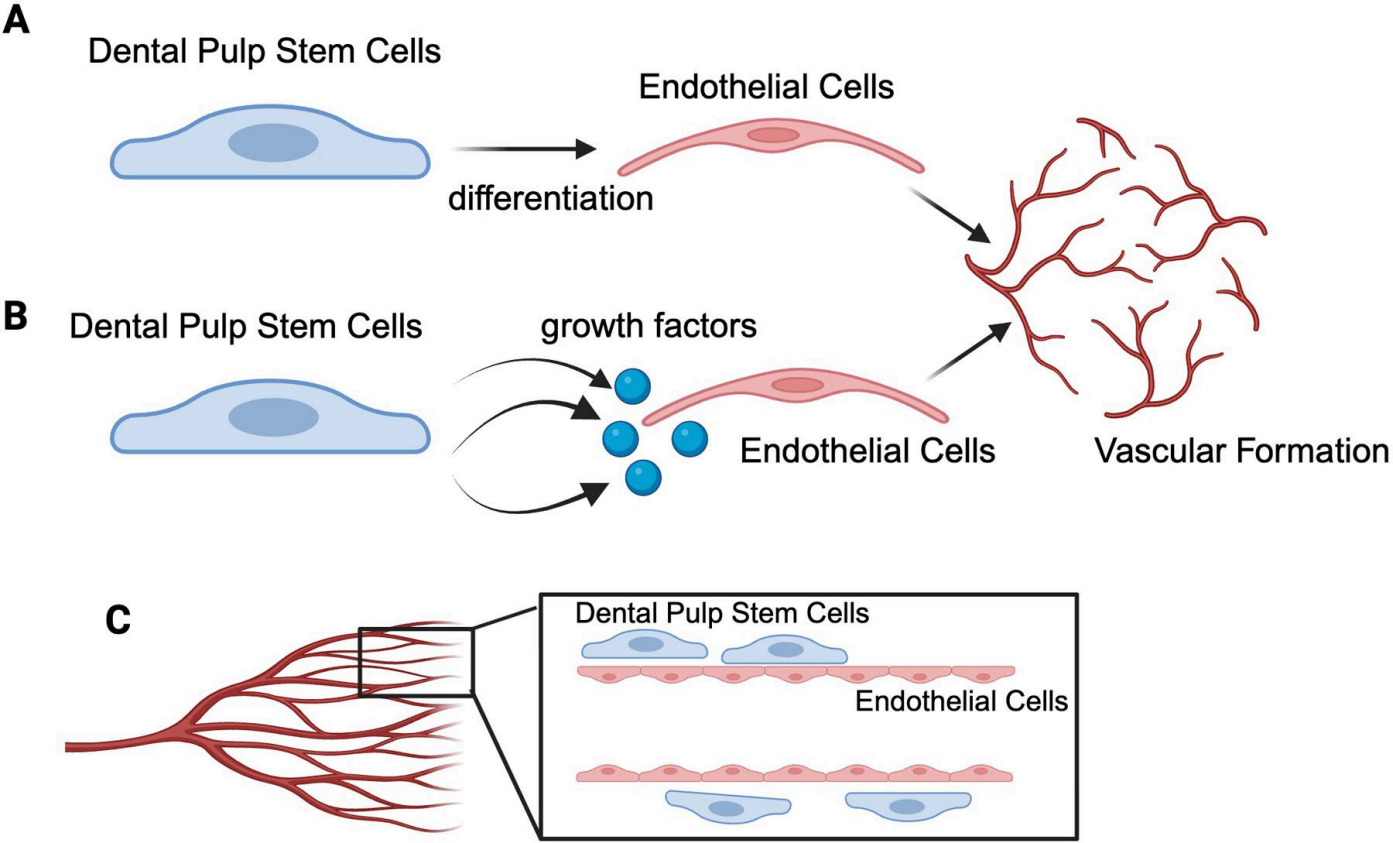
Modulation mechanisms of dental pulp stem cells (DPSCs) in angiogenesis and vasculogenesis. Several potential mechanisms are proposed. First, DPSCs serve as a cell source which can directly differentiate into endothelial cells (ECs) and subsequently form the vascular structure **(A)**. Second, DPSCs modulate angiogenesis by releasing growth factors that act in a paracrine manner to induce vascular formation by ECs **(B)**. Lastly, DPSCs act as pericytes/mural cells, with the ability to stabilise nascent vascular structures, ensuring their integrity **(C)**. Created by biorender.com.

DPSCs modulate angiogenesis and vasculogenesis broadly through several mechanisms (Figure 1). Firstly, DPSCs can differentiate into ECs, suggesting their potential as a cell source for both odontoblasts and ECs to develop vascular networks in dental pulp regeneration (5–7). DPSCs can differentiate into ECs and contribute to the formation of functional blood vessels in immunodeficient mice, mimicking the steps of embryonic vasculogenesis (7–11). Secondly, DPSCs contribute to vessel formation by facilitating EC migration via paracrine action of secreting angiogenic factors and stimulating ECs to form vascular tubes (6, 12–14). Lastly, DPSCs can also act as mural cells to stabilise the nascent vasculature formed.

## DPSCs differentiate into ECs

### VEGF-VEGFR1 signalling

Initial studies on endothelial differentiation have shown that angiogenic factors present in the dentin matrix or released by stromal cells, in conjunction with rhVEGF165, initiate signalling that is sufficient to trigger the endothelial differentiation of stem cells derived from human exfoliated deciduous teeth (SHED), the equivalent of DPSCs in deciduous teeth (5). VEGFR1 signalling has been implicated in the differentiation into ECs, with increased expression of VEGFR2 and CD31 over time (5). Several subsequent studies have shown that DPSCs induced into ECs express significantly higher mRNA and protein expression levels of endothelial-specific genes, including CD31, vWF, VE-Cadherin, VEGFR1, and VEGFR2, and form vascular structures *in vitro* and functional capillaries *in vivo* (6, 15–17). VEGF has been identified as a crucial factor that stimulates the endothelial differentiation of DPSCs, activating several signalling axes, including the Wnt/β-catenin, PI3K–AKT, and MEK ERK1/2 pathways, to regulate the endothelial differentiation of DPSCs (11, 15, 16, 18–20).

### Dentin matrix proteins

Dentin sialoprotein (DSP) and dentin matrix protein 1 (DMP1) are crucial noncollagenous proteins involved in dentin development during tooth formation. Dspp-deficient mice exhibit defective vascular formation in teeth, indicating that Dspp functions in vascular development during dentinogenesis (21). This finding suggests that dentin matrix proteins play a crucial role in regulating the endothelial differentiation. Further, DSP and DMP1 promote the migration and endothelial differentiation of DPSCs. DSP induces endothelial differentiation via the Endoglin–AKT1 signalling axis (22). Additionally, DMP1, in conjunction with a human umbilical vein endothelial cell (HUVEC)-extracellular matrix (ECM) scaffold, promotes the differentiation of DPSCs into an endothelial phenotype (17).

### Genetic modification

Research has explored genetic modification techniques to introduce regulatory signals that promote the differentiation of DPSCs into endothelial cells. DPSCs express various long noncoding RNAs (lncRNAs) during VEGF-induced endothelial differentiation, suggesting lncRNAs may regulate their angiogenic properties (23). DPSCs modified with the lncRNA HRL-SC (Hypoxia-Related lncRNA in Stem Cells) have demonstrated functional characteristics of ECs through the PI3K/AKT signalling pathway (24). In another study, the overexpression of ETV2/ER71/Etsrp, a transcription factor from the Ets family crucial for EC differentiation, has significantly improved the endothelial differentiation of DPSCs by upregulating VEGF receptors, indicating enhanced VEGF signalling during the differentiation process (16). Additionally, a study has shown that vitamin D3, in conjunction with chitosan, collagen, and fibrinogen, induces the transition of DPSCs toward an endothelial phenotype via the HIF-1/IGF-1/VEGF pathway (25). Lastly, Sema4D, via AKT and ERK1/2 signalling (15) and Nell-1 (19), can enhance the differentiation of DPSCs into ECs.

## Angiogenic induction of DPSCs via paracrine action

Angiogenesis is a multi-step process during which ECs detached from an existing vessel proliferate, migrate towards an angiogenic stimulus, and form vascular tubes that are stabilised by pericytes/mural cells. DPSCs are known to secrete many angiogenic factors that can enhance one or more of these steps. Exosomes derived from DPSCs enhance angiogenesis through the Cdc42/p38 MAPK pathway (26). DPSCs are reported to express several angiogenic factors, including VEGF, FGF-2, PDGF, MMP-9, IGF-1, TGF-β, IL-8, and MCP1, with VEGF being the most detected and explored factor (12). Most of the other angiogenic factors are present at levels incapable of exerting significant effects on their own. Therefore, these factors may act in conjunction with VEGF to effectuate their roles. VEGF, which is involved in EC proliferation, migration, and tube formation, exerts these effects by activating the PI3K/AKT and MEK/ERK pathways. DPSC secretion of VEGF increases in response to stimuli such as hypoxia and inflammation (27). VEGF signalling is critical in how DPSCs manage blood vessel formation in prevascularized tissue constructs (13). It has been shown that EphrinB2/EphB4 signalling upregulates VEGF expression in DPSCs and enhances the ability of DPSCs to induce sprouting angiogenesis (28).

DPSCs expressing CD31-/CD146-, along with CD105+ cells with high angiogenic potential, resulted in effective engraftment, enhanced blood circulation, and a significant formation of capillaries (9). It was observed that these transplanted cells were located adjacent to the newly developed blood vessels but did not merge with the pre-existing vessels. They expressed multiple pro-angiogenic factors, including VEGF-A, G-CSF, GM-CSF, and MMP3, suggesting that specific subpopulations of DPSCs could effectively induce angiogenesis and vasculogenesis during pulp regeneration, potentially via paracrine function (9).

### VEGF expression modulation under stress conditions

#### Caries/inflammation

In the context of caries and inflammation, DPSCs can respond to inflammatory stimuli by enhancing their angiogenic capabilities. This is evidenced by increased blood vessel density observed in inflamed dental pulp tissues, such as those from deep caries. VEGF expression is upregulated in pulp tissues from teeth with deep caries compared to that of normal teeth (29). VEGF production in dental pulp cells was enhanced when treated with lipopolysaccharides (LPS) via an sCD14-dependent pathway (30). Another study found that short-term exposure of DPSCs to tumour necrosis factor-alpha (TNF-α) upregulates the VEGF expression and NF-kB signalling (31). VEGF both boosts vascular permeability and aids the chemotaxis of monocytes and macrophages, potentially leading to increased leaky vessels and inflammatory cell accumulation. However, VEGF also promotes the chemotaxis, proliferation, and differentiation of dental pulp cells, which are crucial for recovery during inflamed pulp tissues (32). Hence, VEGF may have a therapeutic role in treating inflamed pulp by promoting angiogenesis and repairing damaged pulp and dentin (30).

#### Hypoxia

Maintaining oxygen homeostasis is crucial for the growth and development of cells and tissues. Hypoxia occurs when the oxygen supply falls below physiological requirements. Notably, hypoxia serves as a vital stimulus for processes essential for tissue regeneration and repair, particularly angiogenesis. HIFs play a pivotal role as transcription factors, not only regulating but also adapting biological responses to hypoxic conditions. DPSCs exhibit a significant response to hypoxic conditions through the upregulation of HIF-1α. HIF-1α activation essentially regulates angiogenesis, as it promotes VEGF expression while enhancing the survival and functionality of ECs (33, 34).

As a promising therapeutic target, HIF has garnered attention for its potential in modulating angiogenesis under various physiological and pathological circumstances. In low-oxygen conditions, HIF-1α attaches to the hypoxia response element (HRE) located in the VEGF promoter, which boosts its transcription—a vital regulatory mechanism in both regular and pathological angiogenesis. Recent studies showed that silencing PHD2 led to a marked stabilisation of HIF-1α levels and an increase in VEGF production in SHED (6). Interestingly, the stabilisation of HIF-1α not only boosted the endothelial differentiation of SHED but also escalated angiogenesis *in vivo* (6). Furthermore, HIF-1α stabilisation enhances SHED survival, improves dentin-pulp tissue formation, and promotes angiogenesis in *in vivo* models of dental pulp regeneration (35). Collectively, these findings underscore the capacity of hypoxia-preconditioned cells to exhibit heightened resistance to adverse *in vivo* microenvironments, while also facilitating a rapid angiogenic response, thus significantly enhancing the efficacy of tissue regeneration therapies.

Accordingly, deferoxamine, a HIF-1 stabiliser loaded into gelatin-based microspheres and encapsulated in a hydrogel composite, has been shown to enhance the angiogenic ability of DPSCs via the expression of VEGF (36). Similarly, conditioned medium from DPSC produced under hypoxic conditions has been proposed as a tool that supports angiogenesis and recruits host blood vessels, suggesting its use to overcome the issues and restrictions associated with cell-based approaches (27).

#### Dental materials, irrigants, and treatment tools

As vital pulp therapy emerges as a regenerative endodontic treatment option, it is crucial to explore the angiogenic effects of existing clinical medicaments, irrigants, and treatment tools. Angiogenic stimulation of DPSCs by the capping materials is a crucial factor that determines the formation of reparative dentin and the outcome of vital pulp therapy. A study has shown that VEGF expression in DPSCs was upregulated by mineral trioxide aggregate, Biodentine, and Emdogain, but not by Ca(OH)_2_, providing insight into the regenerative properties of commonly used pulp capping materials (37). Additionally, low-level laser has been shown to increase the proliferation of DPSCs, elevate the production of VEGF, and enhance angiogenic activity (38).

## Inflammatory/immunomodulation of the DPSC-mediated angiogenesis

The immunoregulatory and angiogenic functions of DPSCs are closely linked and work together, primarily through paracrine signalling and direct cell-to-cell contact, to support tissue repair, regeneration, and immune tolerance in inflamed or injured areas. The interaction between these two roles is essential for effective tissue regeneration, where angiogenesis must happen in a controlled, anti-inflammatory setting. The presence of inflammatory cytokines, such as interferon-gamma (IFN-γ) and TNF-α, stimulates DPSCs to boost their immunomodulatory and pro-angiogenic activities (39). DPSCs release numerous soluble factors, including TGF-β, IL-10, Colony-Stimulating Factor (M-CSF), and Granulocyte Colony-Stimulating Factor (G-CSF), which simultaneously regulate immune responses and promote the formation of new blood vessels. TGF-β and IL-10 are potent anti-inflammatory and immunosuppressive cytokines. TGF-β also plays a role in the later stages of angiogenesis by attracting pericyte-like cells to help stabilise newly formed vessels (27, 40). M-CSF and G-CSF not only encourage monocyte differentiation into macrophages but also shift macrophages toward the anti-inflammatory M2 type, which in turn induces angiogenesis and tissue healing (27, 41). DPSCs/SHED can become pericyte-like cells that physically interact with endothelial cells to stabilise and mature new blood vessels (42, 43). Their strategic positioning helps create an “immune-privileged” area within the healing tissue, further reducing excessive inflammation by inhibiting the formation of leaky vessels. In summary, the immunoregulatory activity of DPSCs creates a conducive, anti-inflammatory microenvironment that is essential for their pro-angiogenic properties to successfully facilitate functional new blood vessel formation and overall tissue regeneration.

Furthermore, DPSCs release exosomes and extracellular vesicles (EVs) that boost angiogenesis under inflammatory stress. A study found that exosomes derived from LPS-stimulated DPSCs promoted the proliferation, migration, and tube formation of HUVECs more effectively than exosomes from non-stimulated DPSCs. This indicates that the inflammatory environment enhances DPSC angiogenic potential, potentially aiding tissue repair and regeneration during inflammation (44). More studies have shown that EVs from periodontitis-affected DPSCs (P-EVs) significantly increased EC proliferation, migration, and angiogenesis *in vitro*, as well as vessel formation *in vivo* (45–47). In this context, miR-378a was upregulated in P-EVs and boosted EC angiogenesis by downregulating Sufu, thereby activating the Hedgehog/Gli1 pathway. This highlights that DPSCs display increased angiogenic properties under inflammatory stress via VEGF-independent pathways as well.

## DPSC recruitment as mural cells for vascular stabilisation

DPSCs are also shown to support blood vessel formation by acting as pericytes/mural cells, stabilising the newly formed endothelial capillaries (48). VEGF/VEGFR2, Ang1/Tie2, and Sema4D-Plexin-B1 signalling have been reported to regulate the recruitment of DPSCs/SHED as functional pericyte-like cells (42, 43, 49–51). Typically, PDGF-BB produced by ECs promotes the proliferation and migration of perivascular supporting cells to the sites of new blood vessel formation. The functional relationships between these supporting cells and ECs occur through both paracrine and autocrine mechanisms, which are crucial for maintaining the stability and function of blood vessels. The dentin matrix contains a higher concentration of PDGF compared to other growth factors, such as VEGF and FGF-2 (52). Additionally, after dental pulp injury, the production of PDGF-AB by ECs is crucial for attracting perivascular supporting cells to the new blood vessels, aiding in their stabilisation and development (53).

More recently, it has been demonstrated that SHEDs express mural cell markers, including NG2, PDGFR-β, α-SMA, and SM22α, under standard culture conditions (42). Sema4D could act on endothelial plexin-B1 to induce the secretion of PDGF, thereby recruiting SHED as mural cells for vascular stabilisation (42, 43). Additionally, TGF-β1 induces differentiation of DPSCs via ALK5 signalling into smooth muscle cells when co-cultured with HUVECs (50). Furthermore, it was demonstrated that in DPSC-HUVEC direct cocultures, Activin A secretion disrupts the VEGF receptor balance in HUVECs, suppressing angiogenic sprouting and enhancing vascular stabilisation (54).

## Angiogenic properties of DPSCs in pulp regeneration

DPSCs exert robust angiogenic and vasculogenic effects via autocrine and paracrine interactions, and also differentiate into ECs and mural cells to form and stabilise the vasculature. While a subpopulation of DPSCs can differentiate into ECs, a combination of these cells with their non-differentiated counterparts may be used to enhance angiogenesis during pulp regeneration (55).

In engineering pulp tissue constructs, building pre-vascular networks that can be anastomosed upon implantation *in vivo* is a promising approach to achieve timely vascularisation, which is facilitated by VEGF secreted by DPSCs (13). Accordingly, it has been demonstrated that the simultaneous overexpression of VEGF and SDF-1α in DPSCs can significantly increase the size of pulp-like tissues and vascular networks during pulp regeneration (56).

One limitation of utilising the angiogenic properties of DPSCs is the need for a large population of cells for clinical use. As with the expansion of passages leading to impaired regenerative potentials, a study has proposed rejuvenating older DPSCs by overexpressing chromobox protein homolog 7 (CBX7). The results have shown that this approach has led to an upregulated expression of VEGF, resulting in an increased number of capillary-like structures and enhanced EC migration. Furthermore, Cu2+ has been proven to achieve CBX7 overexpression in DPSCs through the initiation of the HIF-1α-CBX7 cascade (57).

Pulp revascularisation is a regenerative endodontic procedure that has already reached the clinic. However, the development of scaffold materials that can be clinically used and enhance the angiogenic properties of stem cells is required. In this attempt, a study has synthesised a mineral trioxide aggregate (MTA)-based poly(ε-caprolactone) (PCL)/chitosan (CS) scaffold and demonstrated that DPSCs can adhere to these scaffolds with enhanced angiogenic properties (58).

## Extracellular matrix (ECM) modulation of angiogenesis

ECM plays an underappreciated role in angiogenesis within the pulp. First, the composition of the matrix can either permit or impede endothelial cell migration and tubule formation (59). For instance, an ECM rich in type I collagen and fibronectin provides a favourable substrate for endothelial cells to adhere and form capillary-like structures. In contrast, an ECM high in mineral content or dense cross-linking might inhibit vascular penetration (60, 61). Second, ECM-bound growth factors have a significant influence on angiogenesis. Decorin and heparan sulfate proteoglycans in the pulp ECM can bind pro-angiogenic factors like VEGF and bFGF, protecting them from degradation and creating gradients that guide sprouting vessels (62).

DPSCs contribute to angiogenesis by secreting angiogenic factors when interacting with ECM. For example, culturing DPSCs on a dentin-derived matrix or in hypoxic ECM-mimicking conditions upregulates VEGF secretion by these cells (28, 48, 63–65). Pre-vascularizing scaffolds by co-culturing endothelial cells with DPSCs within a suitable ECM significantly enhanced the formation of vascular networks and improved pulp tissue survival after implantation (48). Taken together, a pro-regenerative ECM should support angiogenesis through both physical pathways and molecular signals. One promising approach is the use of ECM-based scaffolds loaded with angiogenic growth factors; for example, a decellularised pulp matrix pre-loaded with VEGF and bFGF has been shown to markedly improve revascularisation of an emptied root canal space (66, 67). Moving forward, identifying the ECM components or fragments that most potently stimulate angiogenesis could lead to advanced scaffold modifications, ensuring that engineered pulp tissues quickly establish a blood supply after transplantation or cell homing therapies.

## Role of DPSCs in angiogenesis in other tissue regeneration models

Despite the potential benefits of DPSCs for dental pulp tissue regeneration, DPSCs have been reported to modulate angiogenesis and vasculogenesis across various fields of application. The study shows that endothelial cells derived from DPSCs can form tubular structures at the subcutaneous site in a mouse model (68). Further, injection of endothelial cells differentiated from DPSCs into irradiated submandibular glands in mice shows that the group receiving these cells exhibits a higher average saliva flow rate than the control group (68). Transplantation of endothelial cells differentiated from DPSCs, in combination with human oral mucosa cells, promotes the formation of blood vessels in the skin wound model, as evidenced by increased expression of VEGF, vWF, CD31, and CD34 (69). Regarding the angiogenic-promoting mechanism via the release of angiogenic factors, intramuscular injection of DPSCs in a limb ischemic model promotes capillary proliferation and subsequently restores muscle function (70). The potential mechanism could be that DPSCs release higher levels of hepatocyte growth factor and hypoxia-inducible factor-1α (70). Subcutaneous injection of DPSCs-derived exosomes promotes vascular development and angiogenesis in the murine skin full-thickness wound model (26). This is confirmed by the significant increase in capillary-structure formation and the expression of CD31 in the animals treated with DPSCs-derived exosomes (26). Further, conditioned medium from DPSCs promotes bone formation in the calvarial defect model (71). Interestingly, a significant increase in the angiogenesis marker VEGF is observed, suggesting that DPSCs release cytokines or growth factors that promote angiogenesis, thereby enhancing bone formation (71). Taken together, the mechanisms by which DPSCs promote vasculogenesis and angiogenesis are standard not only in dental pulp tissue regeneration but also in general tissue regeneration.

## Conclusion

DPSCs modulate angiogenesis through several mechanisms: direct differentiation into ECs, paracrine release of angiogenic growth factors, and acting as mural cells to support the neovascular structure. In addition, the interaction of DPSCs with ECMs, both in physical and biological aspects, regulates the angiogenesis process. The research findings on the angiogenic regulation of DPSCs could be effectively used in developing strategies that combine pro-angiogenic scaffolds and signalling molecules to overcome challenges in pulp regeneration.
